# Access to Healthcare, HIV/STI Testing, and Preferred Pre-Exposure Prophylaxis Providers among Men Who Have Sex with Men and Men Who Engage in Street-Based Sex Work in the US

**DOI:** 10.1371/journal.pone.0112425

**Published:** 2014-11-11

**Authors:** Kristen Underhill, Kathleen M. Morrow, Christopher M. Colleran, Richard Holcomb, Don Operario, Sarah K. Calabrese, Omar Galárraga, Kenneth H. Mayer

**Affiliations:** 1 Yale Center for Interdisciplinary Research on AIDS/Yale Law School, Yale University, New Haven, Connecticut, United States of America; 2 Centers for Behavioral & Preventive Medicine, The Miriam Hospital, Providence, Rhode Island, United States of America; 3 Project Weber, Providence, Rhode Island, United States of America; 4 School of Public Health, Brown University, Providence, Rhode Island, United States of America; 5 Yale School of Public Health, Yale University, New Haven, Connecticut, United States of America; 6 The Fenway Institute, Fenway Health, Boston, Massachusetts, United States of America; UNSW Australia, Australia

## Abstract

**Background:**

Pre-exposure prophylaxis (PrEP) is a promising strategy for HIV prevention among men who have sex with men (MSM) and men who engage in sex work. But access will require routine HIV testing and contacts with healthcare providers. This study investigated men’s healthcare and HIV testing experiences to inform PrEP implementation.

**Methods:**

We conducted 8 focus groups (n = 38) in 2012 and 56 in-depth qualitative interviews in 2013–14 with male sex workers (MSWs) (n = 31) and other MSM (n = 25) in Providence, RI. MSWs primarily met clients in street-based sex work venues. Facilitators asked participants about access to healthcare and HIV/STI testing, healthcare needs, and preferred PrEP providers.

**Results:**

MSWs primarily accessed care in emergency rooms (ERs), substance use clinics, correctional institutions, and walk-in clinics. Rates of HIV testing were high, but MSWs reported low access to other STI testing, low insurance coverage, and unmet healthcare needs including primary care, substance use treatment, and mental health services. MSM not engaging in sex work were more likely to report access to primary and specialist care. Rates of HIV testing among these MSM were slightly lower, but they reported more STI testing, more insurance coverage, and fewer unmet needs. Preferred PrEP providers for both groups included primary care physicians, infectious disease specialists, and psychiatrists. MSWs were also willing to access PrEP in substance use treatment and ER settings.

**Conclusions:**

PrEP outreach efforts for MSWs and other MSM should engage diverse providers in many settings, including mental health and substance use treatment, ERs, needle exchanges, correctional institutions, and HIV testing centers. Access to PrEP will require financial assistance, but can build on existing healthcare contacts for both populations.

## Introduction

Oral antiretroviral pre-exposure prophylaxis (PrEP) is safe and effective for HIV prevention [1]–[5]. The US Centers for Disease Control and Prevention recently released guidelines for clinical providers regarding PrEP implementation [6], augmenting prior interim guidance [7]–[12]. Among populations at risk for HIV infection, the availability of PrEP will depend in part on access to HIV testing and clinical providers [13]. PrEP prescribers in the US have included nurse practitioners, physician assistants, and physicians trained in family practice, internal medicine, emergency medicine, and infectious diseases [14], [15]. But uptake has not been rapid [15], so in order to identify PrEP implementation barriers, we need to understand where and how at-risk individuals encounter healthcare professionals. Where PrEP implementation guidelines recommend oversight by physicians and other prescribers, access to providers and ability to discuss PrEP with doctors may be key concerns among individuals considering PrEP use [16]–[20].

Ability to pay for PrEP prescriptions, clinical visits, and other components of PrEP care is another central concern. In the US, most individuals continue to obtain health insurance through employment benefits, with smaller proportions obtaining insurance through individual coverage and public insurance programs with defined eligibility criteria [21]. Although estimates vary, the approximate national average cost for ensuring a single individual through employer-based health insurance was $5,384 in 2012, of which the employee paid approximately $1,118 [22]; for individuals purchasing private insurance on the individual market, 2010 data suggest a monthly average premium of $215, yielding an annual cost of $2,580 [23]. In Rhode Island, where we conducted this study, these annual out-of-pocket premium expenses reached $1,335 for individuals insured through employers and $4,128 for those insured through the individual market [22], [23]. Access to both private and public insurance is now growing under the 2010 Patient Protection and Affordable Care Act, particularly in states that expanded the Medicaid program, which offers coverage to low-income individuals [24]. Several prior studies, however, have documented above-average proportions of uninsured individuals among populations at elevated risk for HIV infection, including men who have sex with men [25], [26]. The full cost of tenofovir with emtricitabine (TDF-FTC) for PrEP in the US is approximately $17,000 per year for the medication alone [27]; the total costs of PrEP per individual per year including laboratory services and professional fees approach approximately $18,000 (not including any treatment for side effects). Even among insured individuals, coverage for PrEP may be incomplete, and cost-sharing requirements such as co-pays or co-insurance may limit access [27], [28]. Although the manufacturer of TDF-FTC has instituted a medication assistance program, the effect of this program on access to PrEP is still under study [27].

MSM are a high-priority population for PrEP, given that MSM transmission is associated with the majority of new US infections [1], [29]–[32]. Research is beginning to explore healthcare access among MSM in light of specific PrEP implementation needs [33], as incomplete access to HIV testing and clinical care may inhibit PrEP uptake in the US setting [29], [34]. These impediments may add to other barriers to PrEP use, such as low perceived need or concerns about side effects, drug resistance, efficacy, adherence demands, and behavioral impacts [19], [35]. Suboptimal access to healthcare and HIV prevention services among MSM has been linked to distrust of providers, difficulty disclosing MSM status, minority race and ethnicity, poverty, young age, unknown HIV status, and experienced and internalized homophobia [36], [37]. Reluctance to disclose same-sex sexual behavior to providers may also create barriers to PrEP uptake [26], [38].

Among MSM, male sex workers (MSWs) have specific HIV prevention needs due to elevated numbers of partners, condomless sex, poverty, substance use, prevalence of other sexually transmitted infections (STIs), experiences of violence, difficulty negotiating condoms with clients, sex with male and female partners, low rates of behavior disclosure to clinicians, and difficulty accessing medical care [39]–[47]. Social marginalization and stigma exacerbate barriers to accessing social support, medical care, and HIV prevention services [40], [47]. Sex work itself is associated with additional risk factors, including substance use, unemployment, mental health problems, and childhood sexual abuse [43], [48], [49]. Risks for HIV and other STIs are particularly acute among street-based sex workers compared to MSWs who meet clients in other venues, such as the Internet or escort agencies; for example, compared to their Internet-based counterparts, street-based male sex workers have reported higher rates of HIV risk behavior, lower education, greater unemployment, lower fees for sexual services, more sex work to meet survival or substance use needs, and less negotiation power to refuse condomless sex [43]. Prevalence estimates of HIV among MSWs often exceed estimates among other MSM, including in North America, where studies have documented prevalence of 5–31% [47]. Sex work is illegal and highly socially stigmatized in most of the United States, including Rhode Island [50], [51], where we conducted the present study. Although there have been calls to investigate the potential implementation of PrEP among male sex workers [47], [52], little is known about PrEP acceptability among any subgroup of MSWs, including street-based sex workers.

To improve PrEP implementation, an in-depth understanding of how MSM and MSWs access healthcare is needed. This study used qualitative methods to solicit detailed narratives from these populations exploring healthcare access, HIV/STI testing, unmet healthcare needs, and preferred PrEP providers. The MSWs recruited for this study were primarily street-based; that is, they met clients on the streets or in adult bookstores, and they worked independently rather than through brothels or agencies.

## Methods

We conducted a two-stage qualitative study in Providence, RI. We identified initial themes using focus groups, and then obtained in-depth narratives in individual interviews. All participants met the following criteria: adult biological male; English-speaking; self-reported negative or unknown HIV status; and self-reported condomless anal sex with a man of positive or unknown HIV status in the past 6 months. None had participated in a PrEP efficacy trial. We recruited participants by direct outreach in entertainment venues, sex work venues, community-based organizations, and clinics, and by advertising in local media serving MSM. We collected data in private rooms in clinics. Participants could enroll in both stages, and a small number (approximately 10) did so. But because we anonymized individual interview procedures, we cannot comment with certainty on the number of overlapping participants.

Stage 1 enrolled 38 men in eight focus groups, with 4–6 men per group; three groups were designed to sample street-based MSWs (n = 16) and recruited participants in street-based sex work venues. All men in these groups were MSM who verbally disclosed sex work. Five groups sampled other MSM without focusing on street-based sex workers (n = 22). Five men in these groups disclosed sex work on our demographic questionnaire, and we counted them as sex workers for analyses; we do not know, however, the venues in which these men met their partners (e.g., on the street, in bookstores, on the Internet). These 5 men were recruited through online sources, a flier, and a referral from another study; the 17 MSM who did not report sex work reported learning about the study through online and print media, fliers in bathhouses, clinics, a needle exchange, and word of mouth. Methods for Stage 1 have been reported elsewhere [53]. Groups occurred in February–June 2012.

Stage 2 enrolled 56 men for semi-structured interviews from April 2013 to April 2014. Thirty-one men disclosed selling sex for money, drugs or other goods in the past 6 months, while 25 did not engage in sex work. Of the 31 MSWs, 24 were recruited in street-based sex work venues; the remaining 7 MSWs were recruited through online media, a needle exchange, friends, and a local newspaper. MSM who did not engage in sex work reported learning about the study through fliers at clinics and other venues serving MSM, online and print media, and word of mouth. We screened 92 individuals for focus groups and 110 individuals for interviews. No participant dropped out of the study, no repeat interviews were conducted, and we did not re-contact participants after data collection. We halted data collection after reaching data saturation for main themes in each subgroup (MSM and MSWs).

All participants completed written questionnaires capturing demographic and behavioral information, including information on sexual behavior, substance use, and the CAGE questionnaire for alcoholism [54]. The questionnaire asked about employment but did not specify whether sex work qualified as employment. Given the large percentage of MSWs reporting unemployment, as well as participants’ statements about engaging in sex work due to inability to find or maintain employment, we believe that men did not perceive sex work to be a form of employment for the purposes of this questionnaire. A primary facilitator led qualitative data collection in focus groups (KU), and a secondary facilitator observed group dynamics and took notes. Each interview was conducted by one facilitator (KU or CC), with debriefing to monitor data saturation. All facilitators had prior experience and training leading qualitative interviews.

Facilitators in both stages identified themselves as non-physician researchers funded by the National Institutes of Health, and without a financial interest in PrEP. We provided participants with information about tenofovir co-formulated with emtricitabine for PrEP (e.g., trial findings, FDA approval, side effects, dosage). Focus groups lasted approximately 2 hours, and interviews lasted 90 minutes. Each participant was compensated $75 total for his time and transportation costs. Focus groups and interviews were guided by written agendas; both included modules on PrEP knowledge, willingness to use PrEP, beliefs and intentions about behaviors associated with PrEP use, and comprehension of messages about PrEP efficacy. Individual interview agendas included separate sections discussing access to healthcare, HIV and STI testing, healthcare discrimination, healthcare needs, and barriers to accessing PrEP. Healthcare access was not a planned section of focus groups, but facilitators probed incidental comments about healthcare and analyzed emergent findings. Emergent findings are particularly robust because they arise from spontaneous comments (unprompted by facilitators). This paper reports results regarding access to healthcare and preferred PrEP providers. Findings on overall PrEP acceptability, PrEP messaging, and perceived healthcare discrimination have been presented in conferences [55]–[59] and are being reported in separate publications.

Sessions were audio-recorded, transcribed, and entered into the NVivo 9 program [60]. We developed thematic codes to analyze focus groups and individual interviews, and we applied codes to each verbatim line of text. Initial themes were derived deductively, based on the planned goals of the interview agenda [61]. In the focus group stage, these included barriers to accessing PrEP; in the interview stage, these themes included access to healthcare, unmet healthcare needs, HIV and STI testing behaviors, and access to PrEP. We then identified additional themes derived inductively from the data. In the focus groups, these included prior experiences with clinical care, health insurance barriers to accessing care, preferred PrEP providers, and reflections on the role of providers in PrEP prescription. In interviews, these themes included perceptions of provider-patient relationships (past, present, and ideal), and preferred PrEP providers. Focus groups were double-coded by two independent coders, with discrepancies resolved by discussion and consensus. Interview transcripts were coded by a single coder, and a subset were double-coded by one of two trained research assistants. We examined all coded text for each theme and compared text across interviews and groups to identify analytical findings, seeking both consensus and points of divergence. The findings of this paper are largely reported according to our deductive, question-based categories, using a positivist paradigm [62], [63]. This approach may also be characterized as “semantic” or explicit thematic analysis; given the descriptive goals of the current report, themes were based on the surface meaning of explicit participant statements [64].

### Ethics statement

We obtained written informed consent for focus groups and verbal informed consent for interviews. The Yale Human Subjects Committee and Miriam Hospital IRB approved procedures for both stages, and we obtained a Certificate of Confidentiality from NIH to protect data obtained during individual interviews.

## Results

Results refer to men who engaged in sex work as “MSWs,” and men who did not as “other MSM,” although all participants are men who have sex with men. Descriptive characteristics appear in Table 1. Subgroups were roughly comparable in age and race, but MSWs reported lower incomes, less education, more unemployment, less stable housing, less access to health insurance and primary care providers, more uncertainty about HIV status, and more use of illicit and injection drugs. MSWs were also less likely to identify as gay and more likely to have had sex with women in the past 6 months.

**Table 1:** Selected Sample Characteristics.

	Focus Group Male Sex Workers (n = 21)	Focus Group Other MSM (n = 17)	Interview Male Sex Workers (n = 31)	Interview Other MSM (n = 25)
**Median age (range)**	38.5 (21–57)	39 (27–61)	32 (22–58)	33 (21–70)
**Race**				
White	81.0%	70.6%	77.4%	76.0%
African American	19.0%	29.4%	19.4%	12.0%
Native American	0.0%	0.0%	3.2%	4.0%
Asian	0.0%	0.0%	0.0%	4.0%
Refused	0.0%	0.0%	0.0%	4.0%
**Hispanic or Latino** *	9.5%	11.8%	9.7%	24.0%
**Housing**				
Homeless	19.0%	0.0%	29.0%	4.0%
Staying with friends/family	33.3%	0.0%	38.7%	24.0%
Renting home/apt	42.9%	94.1%	32.3%	52.0%
Owns home/apt	4.8%	5.9%	0.0%	20.0%
**Income <$12,000 per year** **	61.9%	29.4%	51.6%	16.0%
**Education**				
Did not complete H.S.	28.6%	5.9%	29%	20.0%
H.S. or GED only	38.1%	35.3%	35.5%	28.0%
Some college	23.8%	29.4%	32.3%	24.0%
Completed college	9.5%	29.4%	3.2%	28.0%
**Employment**				
Disabled	19.0%	41.2%	6.5%	12.0%
Unemployed	57.1%	23.5%	67.7%	16.0%
Full-time job	14.3%	23.5%	6.5%	28.0%
Part-time/seasonal job	9.5%	11.8%	19.4%	28.0%
Other	0.0%	0.0%	0.0%	16.0%
**Sexual orientation**				
Gay/homosexual	19.0%	58.8%	12.9%	32.0%
Mostly gay	N/A	N/A	6.5%	12.0%
Bisexual	61.9%	41.2%	41.9%	40.0%
Mostly straight	N/A	N/A	19.4%	8.0%
Straight/heterosexual	9.5%	0.0%	12.9%	4.0%
No Response	9.5%	0.0%	0.0%	0.0%
Other	0.0%	0.0%	3.2%	4.0%
Did not know	0.0%	0.0%	3.2%	0.0%
**HIV status**				
Unknown	38.1%	11.8%	35.5%	16.0%
Negative	61.9%	88.2%	64.5%	84.0%
**Received positive STI** **diagnosis (other than HIV)** **in past 6 m**	23.8%	11.8%	3.2%	4.0%
**Health insurance**				
None	52.4%	29.4%	67.7%	32.0%
Insurance through publicsources	38.1%	41.2%	12.9%	36.0%
Private insurance	9.5%	29.4%	19.4%	32.0%
**Time of most recent** **checkup**				
Past 6 months	N/A	N/A	41.9%	36.0%
7–12 months ago	N/A	N/A	22.6%	20.0%
1–2 years ago	N/A	N/A	22.6%	24.0%
Longer than 2 years ago	N/A	N/A	3.2%	4.0%
Does not know	N/A	N/A	9.7%	16.0%
**Has a primary care doctor** **(PCP)**	N/A	N/A	38.7%	56.0%
**Has a PCP and has** **disclosed** **MSM behavior to the PCP**	N/A	N/A	12.9%	28.0%
**Most recent HIV test**				
Past 6 months	N/A	N/A	48.4%	48.0%
7–12 months ago	N/A	N/A	25.8%	16.0%
1–2 years ago	N/A	N/A	19.4%	16.0%
Longer than 2 years ago	N/A	N/A	3.2%	8.0%
Never tested	N/A	N/A	3.2%	12.0%
**Median number of all sex** **partners (male and female)** **in past 6 m (range)**	13.5 (2–150)	10 (2–60)	9 (2–150)	10 (1–50)
**Had sex with both** **male and female partners** **in past 6 m**	52.4%	23.5%	80.6%	52.0%
**Score of 2 or higher on the** **CAGE questionnaire for** **alcohol use** ***	N/A	N/A	29.0%	32.0%
**Used any drugs** **(other than alcohol) multiple times per week in past 6 m**	N/A	N/A	67.7%	28.0%
**Injection drug use in the past 6 m**	38.1%	5.9%	51.6%	4.0%

*As required by the National Institutes of Health, we collected data on Hispanic/Latino ethnicity separately from data on race, and we report these characteristics separately here. Participants who identified as Hispanic or Latino reported races including White, African American, and Refused to Respond.

**The approximate Federal Poverty Line for an individual ranged from $11,170 in 2012 to $11,670 in 2014.

*** = The CAGE questionnaire is a 4-point scale that assists in making diagnoses of alcoholism. A score of 2 or above suggests that the possibility of alcoholism should be investigated further [54].

N/A = Response option was not offered to focus group participants, or question was not asked of focus group participants. We revised our questionnaire to include more questions and response options for the interview stage.

In the remainder of these results, illustrative quotes are reported in text with anonymized research ID numbers. Numbers beginning “FG” represent participants from focus groups, and numbers beginning with “INT” represent participants from individual interviews. Because focus groups did not include a dedicated section on healthcare access, emergent findings from focus groups are represented by fewer quotes. Each section first considers common themes arising across subgroups, then unique findings for MSWs and other MSM. Men in both subgroups considered dental care to be a form of medical care, so findings on dental care are included here as well.

### Sources and Categories of Recent Medical Care

Demographic questionnaires asked all participants if they carried health insurance, and we also asked interview participants about their last checkup and whether they had a primary care provider (PCP) (Table 1). The larger percentage of sex worker participants reporting a recent checkup may reflect mandatory checkups in correctional institutions and substance use treatment.

#### Common Themes

During qualitative data collection, men in both subgroups reported accessing care most recently through emergency rooms (ERs), substance use treatment or mental health clinics, and community-based clinics serving low-income or homeless individuals. All of these venues were more frequently reported by MSWs compared to MSM. Reasons for selecting particular providers tended to include referrals from friends or family, affordability, familiarity based on past care experiences, and geographical convenience. Most MSWs and almost all MSM reported receiving some type of medical care within the past 6 months. Men in both subgroups reported receiving mental health care, substance use treatment, surgical care, and care for injuries.

*INT134-MSW*: I had insurance and then I lost it. So I just went to a, went [to a community-based clinic] because I knew they took people without insurance.

*FG126-MSW*: I’ve been to–I go on like a year, two years, three years without even going to the doctor. [I’ve been to] emergency rooms. I’ve had tons of stuff like that, detoxes, you know.… But not actually getting like, you know CT scans, like full body–like, you know what I’m saying? Stuff like that, physicals or whatever, you know.

*INT151-MSM*: I only go when I’m sick, pretty much.… I get a physical every year, though.

Common methods of payment included out-of-pocket payment, nonpayment, reliance on ER care, care from free clinics, and public insurance (e.g., Social Security Disability Insurance, Medicaid, or Medicare); these methods were more common among MSWs. Private insurance was more common among other MSM; MSWs with private insurance tended to be covered under their parents’ insurance policies.

*INT121-MSM*: Well, my, with my wife, I just got accepted to her healthcare…. I’ve never had healthcare ever in my life…. I am gonna be making an appointment for to get a full physical and get everything done. [Previously I’ve gone to a hospital because] they have to take ya. If you’re hurt, if I go to the hospital with no insurance and my head’s busted open, they’re not gonna tell me no.

*INT108-MSW*: I’d love to go see a doctor regularly, go to the dentist, you know what I mean, get medical and dental and stuff. [My parents] switched health care and I got kicked off…. And plus, I’m not currently talking to my parents right now.

*FG105-MSM*: Doctors, you can get free. I–I’ve been through the whole gambit. You can get a–you can get a doctor, you can get treatment and prescriptions from doctors for free. There’s doctors. Medical care is, is–is accessible in this country. You don’t have to pay some, some doctor 120 bucks just to say, “Hey, I want this stuff.” *FG107-MSM*: Yeah, for–for a good amount of people but not for everybody…. *FG105-MSM*: Every state has, um, they have free care…. I happen to know physicians myself who you say, “Look, I want this and whatever, write this,” and [he does it] unless he thinks I’m going to die from it…. You can always see a doctor if your, if your–if your income is low and you fit certain criteria…. I’m very resourceful. I’m my own best social worker. But, you know, some people sometimes they may think, “Well geez, I can’t do this.” *FG107-MSM*: [Nods “Yes”].

*FG110-MSM*: I have insurance, but I’ve gotta apply every 6 months for it. You know, I get it through [clinics serving the homeless community]. And, um, I ain’t got [private insurance] or none of that, so it’d be–you know, where I go the most they wanna give you is like ibuprofen or somethin’ like that, you know what I mean?….

The range of experiences with healthcare access and affordability demonstrate that for low-income MSM and MSWs, the ability and resources to navigate often-complex processes of accessing free care and low-cost health insurance are important determinants of access to care. As several participants in both subsamples reflected, free care may indeed be available, but the motivation, ability, confidence, and perseverance needed to access these resources vary widely.

#### MSWs

At their most recent care, MSWs were more likely to report accessing emergency care or routine physical exams linked to incarceration or drug treatment compared to MSM.

*INT121-MSW*: Every time when you get committed to the ACI to do time, everybody, they pull your blood, they run your blood, they give you a TB test.

Facilitator: When was your last interaction with a doctor? *INT150-MSW*: Um getting arrested about a year ago and spending an overnight in the jail. They give you a physical at the jail.

*INT129-MSW*: Um, I don’t have a, I don’t have a, um, like a, a regular physician, so I only go to the hospital for injuries really.… I don’t go just for physicals. I haven’t in years anyway.

*INT103-MSW*: I go to the doctor if it’s an, an emergency, otherwise I fight through it like.

Unique methods of payment in this subgroup included free care from correctional institutions, public grants for drug rehabilitation, and a settlement from a personal injury lawsuit.

*INT130-MSW*: Pay for medical care? What the fuck are you talking about? [Laughter] I don’t pay for shit. Um it’s all, you know state slots…. I’ve never paid for medical in my life and never will.

*INT138-MSW*: I don’t have the money or the health insurance anyway….But I’m sure if something like a heart attack or something happened they’d have to treat you no matter what.

Themes arising in healthcare discussions (which we will examine further in a separate paper) included unwillingness or inability to use non-emergency care, suspicion that doctors’ prescriptions had exacerbated or caused substance use disorders, desire to obtain medical insurance, experiences with medical debt, and resources for accessing free care.

#### Other MSM

MSM who did not engage in sex work were more likely than MSWs to receive routine or specialist care. Unique care venues included urgent care and eye care; MSM also mentioned recent visits to dental offices. Recent medical care included routine primary care, infection treatment, routine dental care, management of chronic health conditions, and routine lab work. Methods of payment frequently included private insurance, although some men reported dissatisfaction with private insurance due to limited provider networks and incomplete prescription drug coverage.

*INT146-MSM*: It–it was, uh, pretty routine for me to see my primary care doctor–every three months because I am under his care for hypertension, blood sugar issues, cholesterol issues, and for um, um, a tumor–a benign tumor that is on my pituitary gland.

*INT118-MSM*: Well, I have a primary doctor and I, I, I banged my knee. So [my most recent care] was just for just a routine, uh, injury that I had to my knee. [I found my PCP] from my doctor, my primary care doctor before that one.… At least 15 years [I’ve had a] a primary doctor…. I have Medicaid and Medicare.

*FG102-MSM*: I try to go for my physical every year. My doctor, you know, I’m supposed to take my vitamins, my fish oil, and you know.

*FG129-MSM*: I have good insurance. I even have a, a private [insurance policy] which picks up the other 20% which I pay for, you know, and–and some of my medications are like $280, $300. And Medicaid–the government made such major cuts that I have no choice but to pay for this and it costs me $200 a year just for this prescription card which is well worth it, you know what I mean? Or I would be in big trouble without it.

Criteria for selecting providers included friendliness toward MSM, speed, and short waiting times.

*INT114-MSM*: A lot of the gay community goes to that clinic [where my PCP works]. That’s how I found out from another person that’s gay to go there…. ‘cause you know word of mouth and everything…. I’m going through hell having no insurance. I had awesome insurance, private doctor, everything. Went from that to going to these government clinics. You see the major difference, but beggars can’t be choosers and at least I’m getting help.

*INT119-MSM*: [My most recent care was a] routine general checkup, general physical exam [at an urgent care center]…. I do [have insurance]…. I do pay a little bit more at the urgent care, but it’s much quicker actually…. [I]f I happen to get sick I would go to the urgent care before I went to like the actual health center. That might, I might save a few bucks.

*FG114-MSM*: I met my doctor in the emergency room stitching me up. I loved this chick. I said, “I’m queer, will you take me?” She said, “I’m a lesbian, yes, I’ll take you,” and I have just been lucky.

*FG109-MSM*: My doctor is not gay friendly and I’m trying to look for a doctor that is.

Themes arising in discussions about healthcare included concern about overprescription of psychiatric and other medications, some men’s lack of need for preventive care, concerns about risks of medical procedures, “informal care” by discussing healthcare problems with friends or family who are clinicians, and the desirability of long-term relationships with MSM-friendly PCPs.

As Table 1 demonstrates, MSM were less likely than MSWs to report unemployment or an income level falling below the federal poverty line for an individual, and more likely to report having a full- or part-time job. This difference in employment status may explain much of the variation in access to private insurance, given that most health insurance in the US system is tied to employment benefits. MSM in both stages of this study also reported accessing insurance through public sources, such as Medicare or Medicaid; navigating these enrollment processes may have been somewhat easier for men in this subgroup due to increased education compared to MSWs. Our qualitative findings demonstrate how differences in health insurance access may be experienced as differences in the quality and type of medical care received, as well as the ability to choose among multiple providers.

### Access to HIV and STI Testing

The demographic questionnaire asked interview participants when they had their last HIV test (Table 1). The greater proportion of MSWs reporting testing in the past year may reflect routine testing in correctional institutions, substance use treatment, and a testing initiative by a local outreach organization.

#### Common Themes

Both subgroups reported frequent and recent HIV testing; most participants in both groups reported testing at least annually for HIV, and some MSM not engaged in sex work reported testing more frequently. In contrast, testing for STIs was infrequent and more common among MSM than among MSWs. Very few participants reported undergoing rectal STI testing.

*FG107-MSM*: I just don’t get HIV testing every three months. I get tested for everything every three months.

*INT143-MSW*: I’m kind of uh, I’m paranoid about [HIV] so I wanna know my status and even though it’s not necessary every three months, if you’re active, every three months, yes, but if you’re not, then no, but I still like to hear that confirmation and know that I’m negative.

*INT115-MSM*: Um I was at, I was at a bathhouse and they offered [HIV] testing so I said okay well I might as well go for it.… [T]hey did like a swabbing in the mouth.… I’m kind of old fashioned in the sense that I’d rather give you my blood and, like, make sure, like, everything’s all right.… [My] new doctor has opened my mind to the fact that you must get the STD panel…. And um it’s a good thing that he did because we found out that I had Chlamydia… and he fixed it right away and everything worked out fine.

Common venues for HIV and STI testing included walk-in anonymous clinics, ERs, primary care settings (more likely among MSM), and correctional institutions (more likely among MSWs). The finding for MSWs is unsurprising because Rhode Island state law mandates HIV testing for any person who is convicted of a criminal offense and committed to the adult correctional institution [65], as well as any person convicted of prostitution [66] or possession of a controlled substance that has been administered by injection [67].

Barriers to STI testing in both groups included the perception that all STIs are symptomatic, lack of concern about STIs besides HIV, and lack of awareness that STIs can increase HIV risk.

*INT148-MSM*: I really don’t have an explanation [for why I haven’t asked for an STD test]. Probably because the, the ones I’m aware of have cures.

Several MSWs and one MSM assumed that they were HIV-negative because their partners had tested negative for HIV; each of these participants believed that if he personally had HIV, his partner would certainly have tested positive, so the partner’s negative test was proof that the participant was HIV-negative as well.

*INT127-MSW*: I didn’t get tested [for HIV], but [my girlfriend] did. She came back negative so and I put my thing [penis] in her and so if something happened she probably would have it and she don’t, so….

*INT153-MSM*: Well, I haven’t had [an HIV test] for, I think it was six months…. But my, my, the partner that I recently had intercourse with had his [test] and he came back clean, so I figured I was clean.

Common motivations for HIV testing included recent or ongoing risk behavior, routine testing habits, and joint testing with partners; common barriers to HIV testing were problems obtaining transportation to testing sites and fear of testing because of concerns that recent risk behavior may have caused infection.

*INT144-MSW*: [Testing for HIV is] a knuckle biter because I’ve put myself in such a risk factor that, like, you never know what’s gonna happen. Even like, being safe and not having as many partners as I had, like just maybe one day it’s just gonna pop up outta nowhere.

*INT103-MSW*: [Getting tested] also depends on, like, my relationship status, you know what I mean. If I’m going to be in a relationship, it would be something that me and my partner will go and get tested.

*FG123-MSW*: You say, “Babe, listen, in order to get past this, let’s go get tested. Once we’re clean, that’s it, we’ll move on.”… *FG126-MSW*: Yeah, good point. *FG123-MSW*: What’s she gonna say? No? If she loves you, yeah, if not then see you later.

*INT102-MSW*: Me personally when I’m in, uh, stable or consistent higher risk situations, I’m less apt to seek out HIV testing due to fear of the results.

*INT151-MSM*: There were times where I felt I, I’d engaged in risky behavior and should get a [n HIV] test, but I didn’t … I didn’t wanna know. I didn’t wanna know…. ‘Cause people remember, especially people my age, people got AIDS, you know, in ten months they were dead, six months they were dead.

No participant reported using a home HIV test, citing lack of awareness, concerns about test validity, and concerns about purchasing a test in public. Although many men in both subgroups were willing to use home testing kits for HIV and STI testing (if available), cost was prohibitive.

#### MSWs

MSWs reported ready access to HIV testing. Although a minority of men reported routine STI testing, most reported no recent STI test, and many reported never knowingly testing for STIs.

*INT117-MSW*: Um to be honest, I don’t even know [the last time I got tested for other STIs]. It mighta been a couple years maybe, but I don’t, um, I never even really considered that. I don’t even, that, that’s something that, um, you know, basically all I’m looking out for is like HIV and everything like that. But the other stuff, um, I haven’t even really considered.

MSWs reported receiving HIV tests in research studies, drug rehabilitation centers, needle exchanges, and street-based outreach. The finding that many MSWs test in drug rehabilitation centers is also unsurprising, as Rhode Island requires that providers offer (non-mandatory) HIV testing at facilities treating people who inject drugs [68]; this requirement may also account for the larger proportion of MSWs receiving recent HIV tests generally. Many MSWs also reported that they did not actively seek out testing, but that they accepted it when offered by providers or outreach workers. Several reported obtaining testing by donating blood for payment, assuming that the donation center would contact anyone testing positive.

*INT105-MSW*: [I]t’s not like I set an actual schedule to do it, it’s just happened to be done in some studies … [or] like I, I’ve gone into needle exchange and they’ve offered HIV testing and I’ll take ‘em up on it.

*INT113-MSW*: I give, um, blood regularly so I get tested that way… [at] a place they pay you…. Of course, you have to lie to them…. I can’t say I have sex with, uh, other men.

Unique motivations for HIV testing included self-care intentions linked to substance use treatment, positive tests among friends or family members, the request of a partner aware of one’s sex work behavior, and rumors that certain sexual partners had an STI. Barriers included lack of concern for health during times of severe substance use, and low prioritization of HIV testing compared to other needs.

*INT139-MSW*: Most people get tested for STDs, HIV, and all that is because someone, one of their friends came up to them, was like, “Oh, well, you’re banging this one, you know, you got this, this and this”.

*INT138-MSW*: People don’t go out and get tested for cancer every three, four months. Oh do I have it yet?…. That’s how I feel about HIV too. it’s just another bad disease and you know I don’t, like I said I don’t go out and get tested for anything, never mind HIV.

*INT107-MSW*: I guess I’m always [at the needle exchange] and I always, I don’t want to stay for the test or whatever it is because, um, it’s like I said, the way I’m living I’m, I guess I, I don’t care…. I, sometimes I, um, I don’t want to get tested because I’m afraid I might have it, I guess.

*FG115-MSW*: Like my girlfriend makes me use a condom now until I get tested again, because she knows last month I was out of the house for two weeks and she knows–she knows when I’m gone for two weeks, she knows what I’m doing. I’m getting high and she knows–she’s not stupid. *FG120-MSW* and *FG115-MSW*: Yeah. *FG115-MSW*: So she knows with that also includes me maybe hanging down at that–at the places where I make money.

Many of the quotations from MSWs in this study demonstrate an awareness of HIV risk linked to sex work, but less concern about HIV risk in intimate partnerships. The unique barriers to STI and HIV testing in this group, such as a lack of concern for health during periods of substance use, may pose similar barriers to accessing other services with meaningful impacts on HIV risk, such as PrEP, post-exposure prophylaxis (PEP), mental health services, or substance use treatment.

#### Other MSM

Among other MSM, routine and recent testing for STIs besides HIV was more frequent, perhaps due to a higher proportion of insurance coverage. A minority of men reported never having tested for STIs.

*INT118-MSM*: I’ve been doing it [getting an HIV test] probably every half a year, but I’m late. My doctor’s always got a place where I can go…. I get it all [tested for other STIs too]. When I go for [my HIV test], I make sure that my doctor puts down the whole thing.

*INT119-MSM*: I never thought to ask [about testing for other STIs]. And I never seen nothing going on anywhere thinking that I had anything. And nobody’s ever, you know, I never, the people that I was with, they didn’t seem [to have anything].

*INT137-MSM*: [I get tested for other STIs] once a year.… Like uh like next time I go for a complete physical I’ll have them test me again for all that…. They do it with the Q-tip and I mean, your rectum and everything.

In addition to the locations mentioned above as common venues, MSM reported obtaining HIV and STI testing in workplaces (for occupational health) and bathhouses. Motivations for HIV testing included concerns about partner fidelity, accepting tests that were offered by providers after disclosure of MSM behavior, the desire to protect partners, the ability to advertise a negative HIV test on dating applications, and the desire to set a good example for other MSM.

*INT123-MSM*: Um as I said, I always go, try to [get an HIV test] like two, three times a year…. I understand about the window period because of my family that had it and my friends that had it. So I understand. I, I get checked.

*FG107-MSM*: I don’t use [condoms]. It’s just me. I–I know myself…. I, you know, that’s why I get tested every three months, not for myself but for my partners because I know, I just … because… I can’t use a prophylastic. If I can’t feel it [sex], it don’t work….

*FG111-MSM*: Personally, I think if you are safe, you’re regularly tested you know, you know your status. I mean, a lot of the [dating] sites now, I mean… they have a space where you can put the date of your last test and–and what your status is, which I think is great if you’re going to be hooking up on, like, online things, you know?

*FG102-MSM*: If you love somebody, like, if you know you want to be with this person, I think if two people want to be together then yes, they–they should go get AIDS tests together.

Barriers to testing for HIV included concerns about non-anonymous testing, being reported to a state registry in the event of a positive test, and concerns about partner notification policies in the event of a positive test.

*INT111-MSM*: Um yeah, even though I have health insurance when I get STD tests I still go to the free clinic because then I don’t have to pay a co-pay and it usually takes less time and I don’t have to make an appointment or anything…. And also my dad told me… “If you’re ever gonna get HIV tested go get an anonymous test at a clinic because if it ends up that you have HIV and they disclose it … and the insurance company finds out they’re not gonna wanna keep you on the insurance anymore.” And I was like, “All right, like that’s legit”.

The unique barriers to HIV testing in this group reflect greater awareness and concern about reporting systems, including the potential insurance implications of a positive test result. Although the Affordable Care Act now limits insurers’ ability to deny coverage in group or individual insurance plans on the basis of pre-existing conditions [69], such as HIV infection, individuals who fear HIV testing on this basis may be unaware of this change in legal rules.

### Unmet Healthcare Needs and Barriers to Care

Men in both subgroups reported unmet healthcare needs. Among MSWs, needs included substance use treatment, mental health care, pain management, primary care, eye care, dental care, prescription drug coverage, STI testing, care for hepatitis C, and care for other chronic conditions including diabetes, asthma, migraines, ADHD, and scoliosis.

*INT143-MSW*: I wanna make sure that I got a clean bill of health, and then because of the drug use I ended up uh, is it contracting disease, hepatitis C–and I want to go through that uh, that process to like keep it dormant and you know. If I had health insurance…I wouldn’t have to worry about those things because I would already have all those services.

*INT149-MSW*: Uh, I need dental care. I need, uh, I do need eye glasses…. Um I need, uh, counseling, like therapists. Um, I do um, medication for my, uh, bipolar, anxiety, PTSD stuff…. I have bad teeth, you know…. I was homeless for a period of time and I just couldn’t maintain.

*INT109-MSW*: Well, there are things that I would like that I don’t have the option because I have no income… like mental therapies.

MSM who did not engage in sex work reported some similar needs; these men were more likely to mention dental and eye care, prescription drug coverage, and care for chronic conditions including insomnia, elevated cholesterol, weight problems, chronic obstructive pulmonary disease, and smoking. Several MSM also reported postponing elective surgery (e.g., back surgery) or preventive procedures (e.g., colonoscopy), reporting concern about risks and pain associated with these procedures.

*INT118-MSM*: I need my prescriptions filled because I am a diabetic…. I take type 2, they call it type 2 diabetes…. And I have high blood pressure… and also my cholesterol.

*INT115-MSM*: I just went yesterday for my dental cleaning… And they, they said I had like, ten cavities…. I need to have those addressed. [My public insurance policy] just doesn’t cover it, you know, and it’s tough.

*INT150-MSM*: The only thing I usually need is detoxification services…. The problem would be having no money.

*INT137-MSM*: I don’t have any medical needs right at this point in time, but as long as I have medical coverage, I mean, if something happens to me I’m covered.

*FG110-MSM*: You know, they’ll give a physical and everything else, they–[but my insurance is] not gonna pay for you medication, so it’s kinda tough for me to get any type of, um, medication, you know what I’m sayin’. Um, I gotta dip in my rent money if I need it…. I have other medical problems. I have arthritic gout and stuff like that, you know. I get flare ups and, you know, bad arthritis and stuff…. If I want to ever get some of them pills [PrEP] it’s–I would probably have to give up my whole rent [to afford it] and I’d be in the street.

Both subgroups mentioned similar barriers to care; these included lack of transportation, unaffordability, lack of medical insurance, limited insurance networks, and inflexible work schedules.

*INT116-MSM*: I need more money for medicine. Like, I don’t have the best insurance in the world…. Right now I need medicine for something and I can’t afford it…. [My insomnia is] really bad and, like, I need medicine for it…. I don’t see much of a point to go to the doctors. I get prescribed medicine and I can’t even afford it.

*INT119-MSM*: Like I would like to have access to better more inexpensive health plans…. Like I already have [private insurance]. I would love to have access to other health plans that might be out there. It’d be like Obamacare [accessing health insurance under the new Affordable Care Act], for example, where it might not be as expensive. That I would like to have access to. That would be something I’d look, you know, would look into actually down the road.

*INT122-MSM*: It seems like everything’s, like, so far away, and the only transportation available for me right now is, is walking. Let’s just face it, nobody wants to walk from here, you know, ten miles to get, you know, to a doctor’s appointment.

*INT140-MSW*: Sometimes you’re scared, you’re like you want to get checked for a certain thing, but you don’t have medical and you know if you’re seen the bill’s going to be so sky high and then it sort of builds up. It’s going to ruin your credit.

*INT122-MSW*: Drug rehabs… are all scattered out in the [towns outside Providence], like nothing’s really anyplace close you know…. [I’ve] had to pay, pay, pay people to drive me out there and it’s difficult, ‘cause you know you actually have to buckle down and say all right, I want this… and you have to make the commitment. And that’s the first step that’s the hardest.

Unique barriers for MSWs included lack of interest in healthcare during times of heavy substance abuse, and fear and anticipated shame of discovering physical damage due to long-term substance use.

*INT106-MSW*: Like, it’s hard to hold onto healthcare coverage and jobs and, you know, keeping appointments to get free care, and stuff like that when you’re gettin’ high.

*INT139-MSW*: I’ve done drugs. I’ve done, you know, my fair share of causing like damage to my body…. And going into a doctor’s office and being like, you know, what’s wrong with my body. “Well, you have a hole in your chest from this.” Just pretty much being scared of the, the outcome of the dealings I’ve done to my body.

When asked about unmet healthcare needs, participants rarely mentioned HIV-specific services such as testing or PEP. Instead, they tended to prioritize care for current conditions causing pain or stress. This finding reflects the complex and multifaceted nature of healthcare needs in the MSM and MSW populations; the extent to which individuals in these groups may value PrEP as compared to other type of healthcare is unknown. These data also reveal that although MSM may have had greater access to private health insurance than MSWs, both groups had important structural barriers to accessing preventive care and treatment for medical issues unrelated to HIV infection. Even men with health insurance reported cost-related barriers to some types of care, particularly related to cost-sharing requirements for prescription drug coverage. These barriers will also be relevant for accessing PrEP prescriptions, as well as the provider visits and laboratory testing needed for PrEP implementation protocols in the US. The fear, shame, and personal barriers that MSWs also reported to seeking care are conceptually distinct and need further attention, as these may not be remedied by improved access to health insurance alone.

### Preferred PrEP Providers

No participant had been offered PrEP by a provider, although a majority of men in both subgroups were interested in using PrEP. Both subgroups reported willingness to accept PrEP from PCPs, HIV treatment specialists, and psychiatrists; several men also recommended linking PrEP referrals to HIV testing. MSWs would also accept PrEP from substance use treatment clinicians and emergency room providers. Most men in both groups would prefer to form a long-term care relationship with a provider before asking for PrEP.

*INT130-MSW*: You wanna have a doctor that knows you [to get PrEP]. Then you can be more honest… He’s there for your wellbeing… hopefully not just to take home a paycheck.

*INT150-MSM*: I mean I would, I would, [if I wanted to get PrEP] it would probably be a primary care physician.

*INT123-MSM*: To get prescribed [PrEP] I might talk to my [psychiatrist] and then talk to my physician. [I’d first talk to] my psychiatric doctor. He could probably point me to the right person that I could speak to.

*INT136-MSW*: I probably would ask my psychiatrist about it and see if she knows more about it and, and if she can help me.

*INT148-MSM*: Oh, absolutely [I would ask my PCP for PrEP]. Yeah, I would not have a problem with that at all with my doctor…. I’d just tell him my lifestyle and, you know, I think it’d be a good, precaution…. I’m sure if I’m like, “Doc, there’s something available that could prevent me from getting HIV,” he wouldn’t think twice. He’d already have his script pad out…. I can say in all honesty, if I thought he was going to say no I would never bring it up.

*INT151-MSM*: I would [trust my PCP to prescribe PrEP]…. I mean I, you gotta have some trust, I mean. Facilitator: Would you trust her expertise enough to go to her [about PrEP]? *INT151-MSM*: Yeah, I would go to her I would hope, but she may not…. I mean you can’t be expect, expect your doctor to know every little, every little thing. [But] I would talk to her.

When participants reported unwillingness to discuss PrEP with a PCP, a central reason was that they would prefer to receive PrEP from an infectious disease specialist with more expertise in HIV-related medications.

*FG106-MSM*: I wouldn’t talk to my PCP about it because he’s just not going to be knowledgeable about it. I– I wouldn’t expect him to…. I’d want to see it through a specialist because it really is a much higher level of knowledge base that they need to know about these medications that the average physician probably isn’t going to have.

*Facilitator*: So if you wanted to go and get a pill to prevent HIV, would, what kind of doctors would you try to go see? *INT139-MSW*: Preferably someone that had a degree in that, that field. *Facilitator*: So someone who does HIV or, like, infectious disease? *INT139-MSW*: Yeah. Someone that has, I’m not going to go see a pediatrician because they give out free pills. Hell no.

Many MSWs and some MSM were willing to discuss PrEP with a non-prescriber (e.g., an HIV tester, social worker, or therapist), who could then refer them to prescribing clinicians.

*INT130-MSW*: You know, like, look at this PrEP thing. You know if, uh, I feel if I went to a detox and said, “Hey, you know, I did this and that and the other thing, and what about this [PrEP]?” I feel that, you know, even if they didn’t know anything about it right then … that they’d go and research it for me and come back and say, “All right,” you know, “this is what I know now,” you know….

Men in both groups who had disclosed gay identity, MSM behavior, or injection drug use to providers suggested that those providers should offer PrEP to at-risk patients, rather than waiting for patients’ requests. MSWs also believed that providers in HIV testing centers, correctional institutions, emergency rooms, substance use treatment facilities, and clinics serving homeless individuals should offer PrEP information to clients.

*FG105-MSM*: I don’t know why my doctor knowing that I’m a gay man, you’d think that they’d say, “By the way, there’s something that [prevents HIV]… you can discuss that with me and maybe we could make decisions”.

*INT130-MSW*: [Doctors at the correctional institution] should be educated about PrEP and have the ability to say, “Okay, this guy kinda fits the criteria”…. [If] I’m the next person that gets you know the physical and the doctors see me, look at my arm [and see track marks from drug use]… and he’s gonna be like, “Well maybe I should let this guy know about [PrEP],” you know.

*INT131-MSW*: If I could get [PrEP] from say, like, an ER doctor? Yeah [I would]. If I had to go to my personal doctor, I probably wouldn’t ask.

One MSM participant had sought PrEP from his PCP, but because the PCP was unfamiliar with PrEP, the participant visited a clinic advertised at a gay pride event. There, he obtained a new PCP who prescribed PrEP. The participant noted that the second doctor was also initially unfamiliar with specific PrEP prescription protocols, but the provider’s awareness of PrEP and cultural competence with MSM were facilitators to a successful clinical interaction regarding PrEP. Another MSM participant noted that if PrEP is like PEP, lack of information among physicians may be an independent barrier to access, even among men who are able to access providers generally.

*FG106-MSM*: Even getting PEP isn’t always easy because not even a lot of doctors seem to be aware of it…. I’ve talked to various doctors about it, and ER doctors, and they’re – they’re like, “Never heard of it.” I took it–I took PEP once, um, for a month and basically was only able to get the ER doctor to give it to me when I said, basically, “I want you to call your immune disease department and tell them as if a nurse had exposure you need to now treat this person.” And, and then the–the immune disease [department] was able to call back and say, “Oh, you need to prescribe this, and this”.

The fact that no men had been offered PrEP to date reflects the low rates of PrEP uptake observed thus far in the US [14], [15]. But these data also suggest that many MSM and MSWs are comfortable with a range of potential providers for initial conversations about HIV prevention, including PrEP. Although several men reported comfort advocating for services such as PrEP or PEP in clinical settings (e.g., seeking out amenable providers, or giving information to providers with less knowledge about PrEP or PEP), other men said they were more likely to identify a current trusted provider as a starting point for discussions about HIV and PrEP. Men who experience barriers to accessing providers, or to developing long-term or trusting relationships with individual providers, may be at a disadvantage in accessing PrEP and other HIV prevention services through either of these pathways. Additionally, some trusted providers (e.g., counselors in detoxification settings) may be unable to prescribe PrEP directly in the US, raising the importance of linkages among clinical services.

## Discussion

We examined access to healthcare, HIV/STI testing, unmet healthcare needs, and preferred PrEP providers among MSWs (primarily street-based) and other MSM in Providence, RI. Similarities emerged across subgroups: MSWs and MSM both reported receiving care in ERs, mental health or substance use clinics, and clinics serving low-income clientele; HIV testing was more frequent than STI testing; unmet healthcare needs included acute and long-term medical care. Like other studies among MSM and sex workers [70], [71], we also identified a range of barriers to HIV and STI testing, including fear, concerns about confidentiality, and structural barriers such as cost. Consistent with prior studies, we found that cost may be a central barrier to PrEP uptake [72], [73], given that men in both subgroups reported an inability to access a wide range of other healthcare services due to cost. Unmet healthcare needs across these subgroups included not only HIV prevention services such as HIV and STI testing, but also more basic healthcare needs such as primary care, the management of chronic conditions, laboratory work, prescriptions, substance use treatment, mental health services, and dental care. Importantly, many individuals may perceive other basic healthcare needs as more urgent or important than access to PrEP; few mentioned HIV prevention services when discussing their healthcare needs. We also found that men in both groups had a range of preferred PrEP providers, including PCPs, specialists, and psychiatrists. Our finding that men in both groups preferred PCPs for PrEP implementation differed from a recent study in San Francisco, which found that MSM often preferred to separate sexual health from primary care [33], but early findings on PrEP implementation in San Francisco also report successful PrEP referrals from nonspecialist providers [31].

We also identified differences between MSWs and other MSM in their recent healthcare experiences, which may influence access to PrEP. MSWs were more likely to access care in ERs, substance use treatment programs, and correctional institutions. Routine or required intake procedures in these settings may account for the slightly larger proportion of MSWs reporting recent checkups and HIV tests. In contrast, MSM who did not engage in sex work were more likely to obtain STI testing and non-emergency care in ambulatory care settings. When discussing unmet healthcare needs, MSWs prioritized substance use treatment and mental health services, while other MSM highlighted dental care, eye care, and prescription coverage.

Although the focus of this research has been PrEP implementation, we note that PrEP may not be the optimal HIV prevention strategy for all MSM or MSWs. Men in these groups have many unmet health care needs and priorities, and PrEP may not meet their most pressing healthcare needs. Some MSM or MSWs may not want to use PrEP, some may be unable to use it due to side effects, or consultation with a provider may suggest that PrEP use is not indicated. For these men, access to other types of healthcare services may still be needed, and providing treatment for substance use and mental health disorders may have an independent impact on HIV risk. Men in these populations may prefer other ways of reducing their HIV risk, such as condom use, post-exposure prophylaxis, treatment as prevention, or other strategies; healthcare providers, health insurers, HIV prevention outreach personnel, and the general community should work to support access to a menu of HIV prevention options, as well as the flexibility for individuals to choose among those options.

Within the broader goal of encouraging access to many HIV prevention options, our findings have specific implications for PrEP implementation. We found extensive gaps in insurance coverage among street-based MSWs and MSM. These cost-related impediments to accessing healthcare occurred not only among men without health insurance, but also among insured men who could not afford co-pays or cost-sharing requirements of private health insurance plans. Lack of health insurance has been correlated with increased condomless sex among MSM [74], demonstrating both the need for PrEP and the need for affordable healthcare in this group. For men who wish to use PrEP, implementation solutions are needed to help individuals navigate the process of accessing health insurance, and among insured individuals, to access assistance with co-pay requirements (possibly through drug manufacturers’ medication assistance programs where available). Effective scale-up of PrEP may also require more general advocacy for expanded health insurance options for low-income individuals, including single childless adults. Although many states have now expanded their Medicaid programs to extend health insurance coverage in this group, individuals may need extensive help to gain awareness of these benefits, to apply for coverage, and to learn to use their coverage to access services after their insurance policies take effect.

Our findings also underscore the need for increased cultural competency and provider training in care for all MSM [38], [75] if PrEP implementation is to be successful. Ideally, PrEP should be part of a comprehensive care package for both MSWs and MSM, which would include substance use and mental health treatment, STI screening and treatment, hepatitis C screening, primary care, and long-term management of chronic conditions unrelated to HIV.

Addressing access barriers is critical for providing PrEP and other HIV prevention services to street-based MSWs. Barriers may include lack of access to primary care, lack of insurance coverage for visits, prescriptions, and lab work; and difficulty maintaining provider relationships due to substance use. But this study suggests that PrEP implementation efforts can capitalize on venues where MSWs already see providers. ERs, substance use and mental health clinics, correctional institutions, and HIV testing centers provide opportunities for PrEP education and referrals. MSWs often reported positive experiences in substance use clinics and mental health care, and they preferred to receive PrEP education in these settings. Although PrEP prescription in these settings may be rare and logistically challenging, men may accept referrals from trusted providers to other clinicians who are more familiar with PrEP. PrEP education for street-based MSWs should clarify that PrEP is not addictive; providers should also inform patients that TDF-FTC for PrEP has few interactions with other drugs, and that hepatitis C is not a contraindication to PrEP use.

MSWs in this study reported lapses in care and difficulty visiting healthcare providers due to substance use, relapse, fear of discovering bodily damage due to substance use, and problems with transportation to appointments. Access to substance use treatment may be a higher priority for many street-based MSWs than access to HIV prevention services, including PrEP; although either may be beneficial alone, combining the implementation of PrEP with substance use treatment may have powerful benefits for this population, particularly if substance use treatment facilitates engagement with the care system as a whole. PrEP clinicians might also use strategies known to improve engagement and retention in HIV care, such as reminder calls and texts, contacting emergency contacts after missed appointments, working with community agencies or peer counselors to reengage individuals who have left care, or implementing supportive services such as transportation assistance [76].

For professionals interested in implementing HIV prevention services, including PrEP, among MSM who do not engage in sex work, barriers to uptake and long-term follow-up are lower but not absent. Lack of insurance coverage, lack of prescription drug coverage, and nondisclosure of risk behavior may still complicate PrEP uptake. Many men in the sample expected their PCPs to know about and actively offer them PrEP after a disclosure of same-sex attraction or sexual behavior. PrEP uptake may therefore be enhanced if providers discuss PrEP with patients in their practices who have already disclosed gay/bisexual identity or MSM behavior. This finding complements a recent study of HIV care providers, which found that patients’ requests for PrEP are a primary motivator for providers to prescribe PrEP [77]. If patients and providers are each waiting for the other to begin a conversation about PrEP–or an even more basic level, to begin a conversation about HIV and other STIs–this may create a barrier to access. Education about HIV prevention options and strategies for effective provider-patient communication are needed for both providers and MSM, in order to encourage both parties to initiate discussions about HIV prevention during clinical visits.

This study is among the first qualitative inquiries into healthcare access, HIV testing, and PrEP provider preferences among street-based MSWs and other MSM. Qualitative methods are ideal for identifying heterogeneous and detailed experiences, including subtle differences between subgroups, and our two-stage approach allowed us to probe unsolicited data about healthcare experiences and preferences in a diverse population of MSM and MSWs. We are among the first to explore barriers to PrEP use among street-based MSWs in the US, a marginalized population at increased HIV risk. Our large sample yielded nuanced data and wide-ranging experiences.

Our findings also have limitations. Because we used anonymous interview procedures, we do not know how many individuals enrolled in both stages of this study; we believe this number to be small based on facilitators’ familiarity with participants. Compared to other PrEP acceptability studies among US MSM [16], [19], [78]–[84], our sample was more likely to be low-income or disabled, non-gay-identified, and white and non-Latino, which may limit generalizability of findings. All data were self-reported and may be subject to recall, self-report, and social desirability biases. Our results may not generalize to other groups of MSM or MSWs, and findings from the MSW subgroup may not apply to MSWs who meet partners in other venues besides streets and adult bookstores, such as through the Internet or brothels.

Future research can build on these findings by using population-based and longitudinal methods to quantify healthcare utilization and testing behaviors, subgroup differences, associations between healthcare access and actual PrEP uptake, and changes in health-seeking or healthcare needs over time. Further studies could also examine the prevalence of testing “by proxy” for STIs and HIV (e.g., by donating blood or extrapolating from a partner’s test results). To facilitate optimal implementation of HIV prevention strategies including PrEP, quantitative methods might also examine men’s preferences for different types of providers and self-reported efforts to obtain PrEP and other services. Research should also obtain qualitative and quantitative data on how providers and patients communicate about PrEP after discussing sexual histories. Settings for this work should include ERs, substance use clinics, mental health clinics, correctional institutions, and HIV testing centers. Integrating these venues into PrEP implementation efforts can advance access for MSM, including men who engage in street-based sex work.
